# Supplementary material for: Determinants of Disordered Eating Behaviours (DEBs) among Adolescent Female School Students in Riyadh, Saudi Arabia: A Qualitative Study
Source: Nutrients. 2024 Jul 2;16(13):2119. doi: 10.3390/nu16132119 (PMC11243036; doi:10.3390/nu16132119)
Supplement: Supplementary file 1 [file nutrients-16-02119-s001.zip › nutrients-3050399-supplementary.pdf]

Interview Guide

First of all, I want to thank you for agreeing to participate in this interview. Then, I want to indicate that we are interested in understanding teenage girls' eating behaviours and the reasons why they engage in such behaviours.

It is important to understand that the whole interview and discussed aspects will be private and confidential, used for research means only, and will not be disclosed to anyone under any condition.

- As a teenager, can you describe typical teenage girls eating patterns/ habits or behaviours?

  Prompts: What about you, can you explain your eating pattern on a typical day?

- How do girls your age feel about their weight and shape? (BMI and shape)

  Prompts: How about your weight and body shape?

- We know that particular disordered behaviours are common among teenage girls your age. Can you give your opinion on that? (disordered behaviours)

  Examples: dieting and food restriction, binging, induced vomiting, consuming laxatives, intensive exercising

  Prompts: This (…) behaviour is practiced by teenagers, do you think it is common in your age group? Do you or your friends ever discuss that? How far do you agree with that?

  Prompts: What about you, have you ever practiced this (…) behaviour?

- Why do you think girls your age engage in such behaviours? (predictors and determinants)

  Prompts: How does getting older affect the intensity of these behvaiours?

- Have you ever experienced any of the causes you described?

- Can you think of other causes that might lead to these behaviours?

Thank you for your participation…
